# The role of macrophage migratory behavior in development, homeostasis and tumor invasion

**DOI:** 10.3389/fimmu.2024.1480084

**Published:** 2024-11-11

**Authors:** Michael W. Murrey, Isaac Trinstern Ng, Fiona J. Pixley

**Affiliations:** Macrophage Biology and Cancer Laboratory, School of Biomedical Sciences, The University of Western Australia, Crawley, WA, Australia

**Keywords:** tumor-associated macrophages, motility, invasion, HCK, PI3K p110δ, breast cancer, melanoma

## Abstract

Tumor-associated macrophages (TAMs) recapitulate the developmental and homeostatic behaviors of tissue resident macrophages (TRMs) to promote tumor growth, invasion and metastasis. TRMs arise in the embryo and colonize developing tissues, initially to guide tissue morphogenesis and then to form complex networks in adult tissues to constantly search for threats to homeostasis. The macrophage growth factor, colony-stimulating factor-1 (CSF-1), which is essential for TRM survival and differentiation, is also responsible for the development of the unique motility machinery of mature macrophages that underpins their ramified morphologies, migratory capacity and ability to degrade matrix. Two CSF-1-activated kinases, hematopoietic cell kinase and the p110δ catalytic isoform of phosphatidylinositol 3-kinase, regulate this machinery and selective inhibitors of these proteins completely block macrophage invasion. Considering tumors co-opt the invasive capacity of TAMs to promote their own invasion, these proteins are attractive targets for drug development to inhibit tumor progression to invasion and metastasis.

## 1 Introduction

Tumor-associated macrophages (TAMs) are now widely understood to play a range of mostly deleterious roles in cancer progression. In doing so, TAMs recapitulate behaviors of normal macrophages during embryogenesis, homeostasis and repair. Macrophage behaviors such as growth factor secretion, immune regulation, extracellular matrix (ECM) remodeling and guidance of other cells in developing and healing tissues are subverted by cancers to encourage their growth and dissemination (1, 2).

While macrophages have long been known for their phagocytic and host defense capacities, more recently we have come to understand that they have many non-immune roles, some common to all macrophages and some highly specific to their tissue of residence (3–5). These tissue resident macrophages (TRMs) integrate tightly into all tissues with their heterogeneous phenotypes reflecting different environments and demands. TRM specification begins very early in embryogenesis soon after macrophage precursors (pMacs) migrate into developing organs and differentiate in response to local cues (6). Differentiated TRMs also migrate within tissues during development to guide formation of structures such as the mammary gland ductal network (7). In adult organisms, TRMs patrol their local territory to maintain tissue homeostasis and initiate wound repair by either migrating through or extending long dendrites or shorter finger-like pseudopodia into tissue structures (8, 9). Many of the same mechanisms regulating interstitial migration also control dynamic cell projections in macrophages, which express a unique set of motility molecules for this purpose (9, 10). In invasive cancer, the migratory activity of TAMs is hijacked by tumor cells with TAMs guiding tumor cells out of the tumor and into surrounding tissue, thereby recapitulating embryonic TRM behavior (11, 12).

Many excellent reviews have recently been published on the general biology of TAMs in cancer, including a masterful historical overview of TAMs and their many roles in cancer promotion (2). Rather than undertaking a comprehensive overview of how macrophage behaviors are subverted in cancer development and progression, this review examines the role of macrophage motility in normal development and homeostasis and, with a particular focus on the mammary gland, how cancers co-opt this core function to enable local tumor invasion, which leads to distant metastasis.

## 2 Macrophage biology

The mechanisms by which TRMs contribute to tissue development and homeostasis indicate how TAMs contribute to tumor invasion and metastasis. The need for macrophages in normal development was first revealed by the discovery of multiple congenital abnormalities in organisms lacking expression of either the primary macrophage growth factor, colony-stimulating factor-1 (CSF-1), or its receptor (CSF-1R) (13−16). Macrophages were subsequently shown to colonize embryonic tissues very early to help shape organogenesis and then help maintain tissue homeostasis and restore it after various disturbances (3, 5, 17). In response to local cues, newly arrived TRMs functionally integrate with parenchymal cells to carry out both common core housekeeping functions and highly tissue-specific functions. TRMs make up 8-18% of tissue mass in adult tissues (18). After tissue-specific adaptations, TRMs can undergo additional phenotypic changes in response to perturbations such as injury, infection and disease. In other words, macrophages are chameleon-like in their ability to respond to both short and long term cues in their host tissue. With this finely tuned responsiveness to the local environment, it is not surprising that macrophages are co-opted in a number of ways by disease processes, including cancer.

### 2.1 Macrophage ontogeny

Before fate mapping approaches revealed that macrophages arise from several distinct hematopoietic origins in the embryo, all macrophages were thought to be derived from pluripotent hematopoietic stem cells (HSCs) that differentiated into progenitor cells of the mononuclear phagocytic lineage under the influence of a cocktail of hematopoietic factors, CSF-1 being the most important (19, 20). According to this model, highly proliferative progenitor cells in the bone marrow differentiate into circulating monocytes that enter tissues where they differentiate into macrophages. Once *in situ*, macrophages were considered incapable of further proliferation and were replenished by incoming monocytes (19). However, over the last 15 years, fate mapping studies have revolutionized our understanding of macrophage biology, using lineage markers of macrophages or their progenitors to demonstrate that embryonic TRMs arise from non-monocytic yolk sac macrophage progenitors and fetal liver-derived monocytes (2, 6, 21−23). These embryonic TRMs are long-lived and proliferate locally to maintain numbers (22, 24). Indeed, some TRMs, notably microglia and Langerhans cells, rely entirely on life-long self-renewal although they can be replaced by monocytes if profoundly depleted (23, 25, 26). Distinct TRM populations in the same organ can demonstrate different replacement kinetics with embryonically-derived Kupffer cells replaced by self-renewal in the healthy liver while liver capsular macrophages are monocyte-derived (5, 17, 27). In contrast, macrophages in the gut and dermis, which undergo rapid turnover, rely on circulating monocytes to maintain their numbers (28, 29). In a fitting twist that exemplifies the developmental role of macrophages, embryonic macrophages shape the architecture of the hematopoietic niche for HSCs in the fetal liver and the adult bone marrow such that their depletion leads to premature differentiation of HSCs (30).

**Figure 1:**
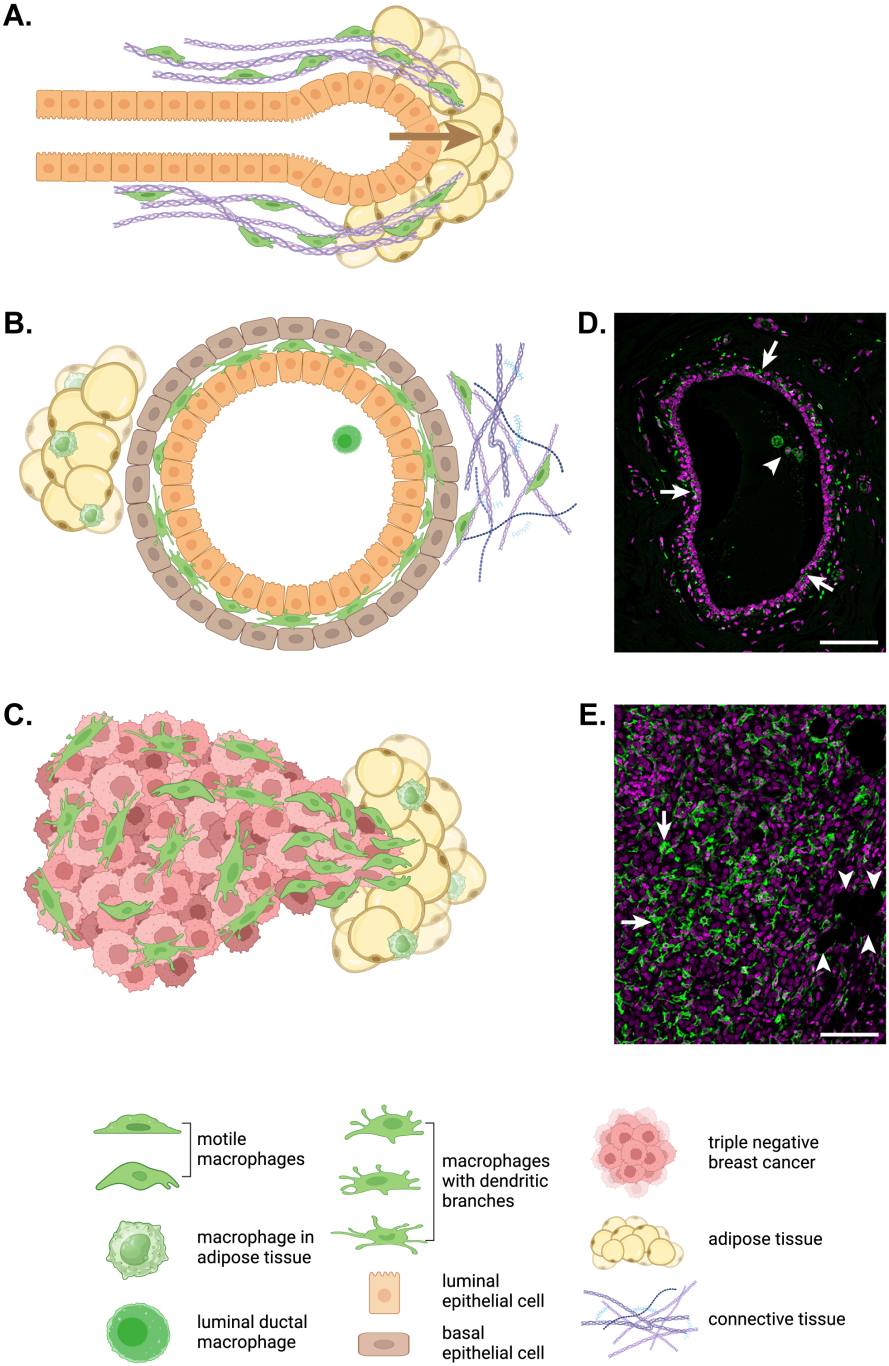
Macrophage motility in development, homeostasis and breast cancer. **(A)** In the developing mammary gland, terminal end bud outgrowth and elongation into the mammary fat pad is guided by macrophages (green) that move along and remodel collagen fibrils surrounding the developing duct. **(B)** In the adult mammary gland, elongated and branched macrophages (green) are found intercalated between the luminal and basal epithelial layers of mammary ducts embedded in a mix of adipose and connective tissue with their own tissue resident macrophage populations. While ductal macrophages do not migrate through tissue, their dendritic branches routinely patrol the ductal epithelium (33). **(C)** In invasive triple negative breast cancer, macrophages (green) accumulate in large numbers particularly at the invasive front. For the immunofluorescent immunohistochemistry images of a normal human mammary duct **(D)** and human triple negative breast cancer **(E)**, ionized calcium binding adaptor molecule (IBA)1+ macrophages are shown in green and nuclei are magenta. Arrows indicate ductal macrophages and the arrowhead points to a group of luminal macrophages in **(D)**. In **(E)**, arrows indicate TAMs and arrowheads point to adipocytes at the invasive front. Scale bars represent 100µm. The schematic diagrams in this figure were created in BioRender.com.

Unlike most organs, development of the mammary gland largely occurs postnatally (31). A study that used CD11b as a marker of mammary gland macrophages revealed persistence of fetal macrophages in the stroma of the postpubertal mammary gland (32). However, a CD11b-/Cd11c+ ductal macrophage population was recently identified lying between the luminal and basal ductal epithelial cells in mouse mammary ducts (33, 34). The same intraductal population of TRMs is seen between the epithelial layers in human mammary ducts and their branched morphology is very different to that of the large, circular macrophages seen within the duct lumen (**Figures 1B, D**). Compared to stromal macrophages, ductal macrophages form a small proportion of TRMs in the virgin mouse mammary gland but expand 40-fold during pregnancy, through both local proliferation of embryonic macrophages and recruitment of bone marrow-derived monocytes, before decreasing to baseline numbers in involution (33). Lineage tracing was used to show that initially both stromal and ductal TRMs are embryonically derived with stromal macrophages slowly replaced over time while embryonic ductal macrophages are largely replaced by monocyte-derived macrophages during puberty after which they self-renew (33, 34). In general, however, circulating monocytes do not act as a supply reservoir for most TRM populations in steady-state. Rather they are recruited in large numbers to sites of inflammation, infection or injury then typically disappear unless inflammation persists (5, 27). As ‘wounds that never heal’, tumors attract and retain monocyte-derived TAMs, often in huge numbers if the tumor cells secrete CSF-1 and other macrophage or monocyte chemokines such as CCL2 (35, 36).

### 2.2 TRM plasticity: specification, activation, morphology and function

While lineage tracing and single cell technologies have confirmed the extraordinary heterogeneity of TRMs, striking morphological differences between tissue-specific macrophages such as Kupffer cells, microglia and alveolar macrophages had long been recognized (37). Morphologically disparate TRMs also exist within organs, for example ramified microglia, spindle-shaped meningeal macrophages and stellate choroid plexus macrophages in the brain, reflecting the niche-specific demands placed on TRMs (38, 39). As noted earlier, embryonic tissues are colonized by pMacs, which express a set of core macrophage genes under the influence of CSF-1 and the macrophage lineage-determining factor PU.1 (5, 6). As pMacs migrate into tissues, they rapidly differentiate into tissue-specific TRMs in response to local cues, the process driven by upregulated expression of tissue-specific TRM lineage determining factors (6, 40–42). Hence, Kupffer cells upregulate ID3, microglia SALL1 and alveolar macrophages PPARγ with a host of other lineage determining factors driving specialization in other TRM populations (5, 6, 39, 43).

After tissue specific adaptation, macrophages can undergo additional phenotypic changes in response to exposure to external cues such as cytokines, microbial products and other modulators (44). For some time, these changes were believed to occur in a binary fashion with a ‘classical’ or M1 phenotype developing in response to interferon (IFN)γ or toll-like receptor (TLR) ligands and an ‘alternatively activated’ or M2 phenotype arising after exposure to interleukin (IL)-4, which was thought to reflect the transition from inflammation to repair (45). However, this is now known to be a very simplistic representation of the full range of macrophage activation states in response to a panoply of modulators. Transcriptional analyses of human macrophages activated *in vitro* by a diverse range of stimuli or in different murine TRMs *in vivo* indicate that many distinct gene expression changes occur between each macrophage population (40, 46). Indeed, the full spectrum of macrophage activation states is very complex and appears to be of limited use in the context of human health and disease (1, 2). Yet the oversimplified classification of TAM activation phenotypes into M1-like or anti-tumoral and M2-like or pro-tumoral unfortunately lingers despite strong evidence of TAM phenotypic diversity in a range of different cancers such as breast cancer and glioblastoma (47–49).

Morphological changes are central to TRM differentiation and specialization. Microglia form a highly ramified, regularly spaced network in the brain (37, 38). Several other TRM populations create similarly complex tissue surveillance networks such as epidermal Langerhans cells whose dendrites and migration towards lymph nodes resulted in their misidentification as dendritic cells for decades (50). Early immunohistochemical studies also demonstrated highly dendritic morphologies in bone marrow stromal macrophages (37). Similar if less complex membrane extensions are seen in other TRMs such as Kupffer cells, which use finger-like extensions to sample liver sinusoidal fluid, and lymph node subcapsular macrophages, which extend fingers upwards into the subcapsular space to capture antigens as well as long branches downwards into underlying follicles to interact with B cells (51, 52). Even yolk sac pMacs have a stellate morphology (18). While it is difficult to observe dynamic behavior of TRM dendritic networks deep in tissues, high resolution live imaging in the mammary gland has shown that mammary ductal macrophages move their dendrites constantly to survey the entire ductal epithelium within a two hour cycle (33). Similarly ramified ductal macrophages can be seen lying between the luminal and basal epithelial layers of the collecting ducts and in lobules in human breast tissue. Dynamic TRM responses to injury have also been captured by intravital imaging in the peritoneum (53).

However specialized, TRMs still carry out core macrophage functions such as phagocytosis and immune surveillance (3). Migration is also an essential core function, which is used by pMacs to colonize embryonic tissues and by differentiated TRMs to guide tissue morphogenesis (9). Although mature TRMs, considered by some to be sessile, may no longer move through tissues routinely, dynamic dendrite movement in interstitial or sinusoidal spaces is unceasing and, when tissue injury occurs, TRMs can extend pseudopods to cloak microlesions and limit inflammation or move into larger wounds to orchestrate repair (33, 53, 54). Thus, TRMs retain the capacity for interstitial migration, which can be coopted to facilitate tumor invasion.

The molecular mechanisms that underpin formation of protrusive membrane structures in macrophages such as the leading edge of a migrating cell, a phagocytic cup or a probing dendrite are similar and involve actin polymerization and coordinated formation of specialized adhesions to enable rapid responses (9, 55). TRMs selectively express a complex array of adhesion and actin cytoskeletal remodeling proteins to enable them to extend, maintain and restructure these processes (9). Moreover, TRMs are embedded in ECM and express a huge number of matrix metalloproteinases (MMPs) and cathepsins to enable protease-dependent mesenchymal migration (10, 56). CSF-1R signaling is essential for the expression of this unique set of motility and matrix degrading proteins as evidenced by the myriad changes in expression of genes regulating adhesion, actin cytoskeletal remodeling and matrix degradation seen with CSF-1-induced differentiation of non-adherent progenitor cells into mature, adherent macrophages (9, 10). The dependence on CSF-1R signaling for macrophage motility is underscored in zebrafish with an inactivating *Csf1r* mutation. Yolk sac-derived macrophage progenitors in the mutant zebrafish are unable to migrate into the cephalic mesenchyme to become microglia (15). Hence, to acquire full tissue-specific functionality, TRMs require CSF-1R signaling as well as niche-specific signals.

### 2.3 CSF-1R signaling to macrophage morphology and motility

CSF-1R signaling is essential for TRM survival and self-renewal as well as differentiation, morphology and function, as demonstrated by the almost total depletion of most TRM populations following administration of a CSF-1R blocking antibody (20, 57). CSF-1 was originally considered the only CSF-1R ligand but the more severe developmental abnormalities of the CSF-1R-deficient mouse led to the discovery of an additional ligand, IL-34 (16, 58). However, CSF-1 drives the expansion of the majority of TRMs required for normal development and homeostasis, microglia and Langerhans cells excepted (14, 59). The striking effects of CSF-1 on macrophage morphology are easily observed in bone marrow-derived macrophages *in vitro*. CSF-1 triggers actin polymerization and adhesion formation to cause ruffling and spreading within a minute followed by further spreading, polarization and finally migration over the ensuing 10 minutes (60–62). The CSF-1R is a class III receptor tyrosine kinase that autophosphorylates multiple tyrosine residues to create binding sites for docking and activation of downstream signaling proteins (20, 63). A macrophage cell line system with individual CSF-1R tyrosine mutants was used to identify two autophosphorylated CSF-1R tyrosine residues, Y721 and Y974, primarily responsible for triggering signals to macrophage motility (62, 64, 65).

Loss of signaling from Y721 in the CSF-1R greatly reduces CSF-1-induced actin polymerization and adhesion formation, resulting in a striking reduction in macrophage motility (62). CSF-1R pY721 binds and activates the class IA phosphatidylinositol 3-kinase (PI3K) to produce a rapid pulse of PI 3,4,5-trisphosphate (PIP3) at the leading edge membrane (62, 66). PIP3 then triggers membrane translocation of pleckstrin homology domain-containing molecules such as AKT/PKB to activate growth, survival and proliferation as well as migration signals (67, 68). The three catalytic isoforms of PI3K, ubiquitous p110α and p110β and hematopoietically restricted p110δ, all of which are expressed by macrophages, have non-redundant biological roles and isoform selective inhibitors indicate that only PI3K p110δ activates CSF-1-induced macrophage motility and matrix degradation signals (68–70). Reflecting the importance of macrophage motility on tumor invasion, PI3K p110δ inhibition also completely blocks co-migration and invasion of co-cultured macrophages and tumor cells in an *in vitro* invasion model (36). Although the precise motility pathways downstream of PI3K p110δ have not been fully elucidated, AKT, Rho family GTPases Rho, Rac and Cdc42, along with Src family kinases (SFKs) regulate actin cytoskeletal remodeling and phosphorylation of adhesion proteins such as paxillin and leupaxin (9, 10). However, because PI3K-activating motility signaling involves bifurcating pathways, direct inhibition of PI3K p110δ is likely to be a more successful strategy to target macrophage motility.

Adhesion and motility in macrophages are also regulated by SFKs (65, 71). Macrophages express no less than five SFKs, each of which has overlapping and unique functions (65). Expression of HCK and LYN increases as macrophages differentiate from non-adherent precursors under the influence of CSF-1 while SRC and FGR decrease (10). CSF-1R pY974-based signaling regulates at least some of these changes as FGR expression is dramatically increased in CSF-1R Y974F mutant macrophages, which spread and move slowly in response to CSF-1 (65). Of the SFKs expressed in macrophages, only HCK and LYN associate with the CSF-1R, HCK in a CSF-1 dependent manner, suggesting it transduces signaling from the activated CSF-1R (65). Confirmation that HCK is the primary SFK transducing the CSF-1R motility signal in macrophages was provided by the observation that macrophages expressing constitutively active HCK move faster, digest matrix more efficiently and encourage greater tumor cell invasion *in vitro* than control macrophages while a HCK selective inhibitor, RK20449, blocks motility, degradation and invasion of both control and constitutively active HCK macrophages (72). Constitutive activation of HCK also drives increased invasion *in vivo* in a gastric tumor model (72). Thus, HCK is an attractive target for macrophage motility inhibition as a therapeutic strategy.

### 2.4 CSF-1R signaling and macrophage motility in the mammary gland

The importance of CSF-1R signaling and macrophage motility is evident in the developing mammary gland. During puberty, mammary epithelial structures called ductal terminal end buds grow into the mammary fat pad then elongate and branch to fill the fat pad with a complex ductal tree (**Figure 1A**) (31). CSF-1-dependent TRMs are recruited in large numbers to the neck of terminal end buds to help guide ductal morphogenesis as ductal length and branching are reduced in CSF-1-deficient female mice while transgenic over-expression of CSF-1 produces increased branching (31, 73, 74). Intravital imaging has shown that mammary gland macrophages associate with collagen fibers found alongside growing terminal end buds and that these macrophages migrate along the fibers and fuse shorter fibers to promote their elongation, thereby shaping ductal outgrowth into the mammary fat pad (**Figure 1A**) (7). Mammary macrophages also shape lobular morphogenesis during the estrous cycle and pregnancy and phagocytose apoptotic epithelial cells during involution (75). There are large increases in ductal TRM numbers during puberty and pregnancy to facilitate these processes (32, 33). Importantly, ductal and not stromal macrophages are thought to be co-opted by tumor cells to become TAMs in breast cancer (33, 34).

## 3 TAMs and tumor progression

Tumors are aberrant organs with their own integrated populations of resident macrophages known as TAMs. Indeed, most solid tumors contain large numbers of TAMs with a high correlation between TAM density and poor outcome in many types of cancers in humans, including breast cancer (76–79). Single cell transcriptomic studies have confirmed both the abundance and heterogeneity of TAMs within and between tumor types in a range of human cancers, including breast cancer (43, 79–82). Consistent with these observations, TAM density is striking highly in triple negative breast cancer, which has the lowest survival of breast cancer subtypes (**Figures 1C, E**) (82, 83). Furthermore, macrophage heterogeneity is increased in tumors compared to nearby normal tissue (81).

It is now well understood that TAMs co-evolve with cancers and contribute to their development and progression in several ways, including support of tumor growth through production of growth factors, promotion of angiogenesis through secretion of pro-angiogenic factors and immunosuppressive effects on the adaptive immune system (2, 49, 84–87). However, perhaps the most lethal contribution TAMs make to tumor progression is their promotion of tumor invasion and metastasis (2, 88−90). Consistent with this notion, TAMs have been shown to accumulate at the invasive front of breast cancers (**Figures 1C, E**) (91).

### 3.1 CSF-1R signaling in TAMs and tumor invasion

The importance of CSF-1R signaling in cancer progression was originally hinted at by the association of high circulating levels of CSF-1 with poor outcomes in breast, ovarian and endometrial cancers and further supported by the co-localization of CSF-1 expressing carcinoma cells with CSF-1R+ TAMs in invasive breast cancer (91–94). Confirmation that CSF-1-dependent macrophages promote tumor progression, particularly to invasion and metastasis, was provided by an experimental model in which the CSF-1-deficient osteopetrotic mouse was crossed with the polyoma middle T (PyMT) mouse, an autochthonous model of breast cancer to produce CSF-1-deficient PyMT mice. Multifocal mammary tumors arise and progress steadily to pulmonary metastasis in the female mice and, while initiation and early progression of mammary tumors are unchanged, late stage progression is slowed and pulmonary metastasis is all but halted in the absence of CSF-1-dependent TAMs (88, 95). Inhibition of pulmonary metastasis is due in part to the failure of tumor cells to disrupt the basement membrane unless macrophages are present (88, 89). This is because TAMs set up a paracrine chemokine interaction with tumor cells to activate tumor invasion via a mechanism of relay chemotaxis (89). Tumor cells secrete CSF-1 and TAMs secrete epidermal growth factor (EGF) to enable both cell types to co-migrate along collagen fibers in an alternating fashion (11, 89). Notably, either CSF-1R or EGFR inhibition signaling completely stop invasion of both TAMs and tumor cells (89, 96).

*In vitro* live imaging of co-cultured mammary tumor organoids and bone marrow-derived macrophages enables a closer examination of relay chemotaxis and reveals that tumor cell invasion from the organoids only occurs after motile macrophages that had previously exited the organoid make contact with the tumor cells to activate their motility then lead them into the surrounding matrix (36). Flow cytometric analysis of the invasive cells revealed a 3:1 ratio of tumor cells to macrophages (36). In this co-invasion assay, selective inhibition of either HCK or PI3K p110δ are equally as effective as CSF-1R inhibition in shutting down macrophage-led tumor cell invasion while macrophages expressing constitutively active HCK promote increase tumor cell invasion (36, 72). Consistent with a requirement for matrix degrading activity in invasive macrophages that lead tumor cells out of tumors, upregulation of cathepsin protease activity increases the invasion-promoting activity of TAMs (97). Underlining the central role of motility in this interaction between macrophages and tumor cells, gene expression studies of co-migrating tumor cells and TAMs show upregulation of motility genes in the tumor cells with upregulation of trophic genes in the already motile macrophages (98, 99). Thus, motile TAMs appear to be an essential component of tumor cell invasion in at least some invasive tumors such as breast cancer. In a form of what has been labeled ‘oncofetal reprogramming’, interstitial migratory TAMs recapitulate the motile and matrix remodeling behavior of embryonic TRMs and activate tumor cell motility to lead them through the basement membrane and into nearby tissue (100). This leads to the notion that not only does TAM-dependent invasive activity lead to metastatic spread due to incidental breaching of blood or lymphatic vessels but, by releasing physical constraints on primary tumor growth, invasive TAMs contribute to primary tumor growth, i.e. invasive growth.

### 3.2 TAM ontogeny and heterogeneity

Until the identification of self-renewing embryonic TRMs, all TAMs were thought to be derived from circulating monocytes. It is important to note, however, that maintenance of TRM populations through self-renewal relies on steady state conditions as perturbations such as extensive tissue injury or experimental macrophage depletion can lead to replacement of TRMs by circulating monocytes that differentiate into TRMs, albeit with distinct phenotypes (2, 6, 23, 101). As flagged earlier, monocytes are also recruited in large numbers to sites of inflammation and, since chronic inflammation is a consistent feature of cancer, it is not surprising that monocyte-derived TAMs are found in large numbers in many tumors (5, 35). However, as TRMs are proliferative, TAMs can also arise from local TRM populations. Consistent with this possibility, parabiotic studies in a mouse model of pancreatic ductal carcinoma showed that embryonic TRM-derived TAMs appear to predominate and they drive the strong fibrotic response so typical of these tumors (102). In contrast, TAMs in spontaneous PyMT mammary tumors are predominantly monocytic in origin (103). Other tumor types display a mix of monocyte-derived and TRM-derived TAMs, for example early non-small cell lung cancers contain mostly TRM-derived TAMs that are gradually replaced by monocyte-derived TAMs as the tumor progresses, and brain cancer (86, 104). Thus, it would appear that monocytes and TRMs account for distinct proportions of TAMs in different mouse models of cancer and these proportions can change over time as the cancers progress (101, 105).

TAM ontogeny in human tumors is more difficult to tease apart for obvious reasons. Nevertheless, a meta-analysis of single cell transcriptomic data from lung, colon and liver cancers and nearby normal tissues demonstrated an increase of monocyte-derived macrophages compared to normal tissue, although TRMs contributed to TAM numbers in liver cancer (43). Consistent with the largely postnatal development of the mammary gland, lineage tracing and other approaches used to map the ontogeny of TAMs in experimental models indicate that TAMs are largely monocyte-derived in breast cancer. In the PyMT mammary tumor model, TAMs are phenotypically distinct from TRMs and are recruited from the bone marrow through tumor cell secretion of CSF-1 and the monocyte chemokine CCL2 (36, 101, 103). Continuous seeding of monocyte-derived TAMs was also demonstrated in three additional models of breast cancer (47). Whether monocyte-derived TAMs also predominate in human breast cancer is currently not clear, due in part to TAM heterogeneity. Nevertheless, it is likely that the majority of TAMs in human breast cancer are monocyte-derived while TRMs contribute to one or more TAM subtypes (106). Metastasis-associated macrophages are functionally distinct from primary tumor TAMs and are also predominantly monocyte-derived and recruited by tumor cell-secreted CCL2 in the PyMT and other models of breast cancer that give rise to pulmonary metastases (2, 107). However, as both monocyte-derived TAMs and TRM-derived TAMs have the capacity to proliferate, it is likely that both populations contribute to the abundant TAMs that accumulate in human tumors and their metastases, with the balance of each contribution differing between tumor types (2).

Just as there are diverse TRMs within individual organs, human tumors contain heterogeneous TAMs. A study comparing breast and endometrial cancer revealed that TAMs within tumors are more diverse than and distinct from TRMs in neighboring normal tissue and also that they differ between tumor types (79). Considerable inter-patient variation in TAM abundance and phenotypes is also seen in individual cases of human breast cancer (81). Similarly, distinct transcriptomic profiles of diverse TAM subtypes can be seen across many different human cancer types (80, 87). Spatial transcriptomics has added yet more complexity to the classification of TAMs and this has been further complicated by evidence that progressive differentiation of TAMs can occur within tumors. For example, invasive TAMs that co-migrate hand in hand with tumor cells out of tumors transition into sessile perivascular TAMs in the vicinity of blood vessels in PyMT breast cancers (108). Similarly, in an orthotopic PyMT model of breast cancer, the adipose tissue-rich environment of the mammary gland induces a lipid-associated phenotype in all TAM clusters whereas this phenotype only occurs in specific subtypes in other cancers (Murrey at al., under review) (82, 109, 110). Thus, the influence of the tumor environment and nearby tissues on TAM phenotypes is critical. Moreover, while transcriptomic analysis might classify particular TAM subtypes as pro-angiogenic or immunosuppressive, other subtypes also express angiogenic and immune suppressing genes.

Despite this heterogeneity, consistent subtypes are found across different tumor types such as angiogenic TAMs that accumulate in hypoxic, necrotic regions and immunosuppressive TAMs that inhibit cytotoxic T cells and NK cells and recruit immunosuppressive regulatory T cells (2, 47, 111). Because of this, two groups recently attempted to develop a consensus nomenclature for TAM molecular subtypes using single cell and spatial transcriptomic data extracted from several pan- cancer data sets. Six subtypes plus proliferating TAMs were identified with broad agreement across four subtypes - interferon-primed/interferon-mediated regulatory TAMs, immune regulatory TAMs, inflammatory TAMs and proangiogenic TAMs – with disagreement on whether lipid-associated TAMs constitute a specific subtype, which may reflect the adiposity of the tumor environment (109, 110).

A couple of additional points regarding single cell studies of TAM heterogeneity deserve consideration. Firstly and relevant to interpretations of mouse models of cancer, TAM heterogeneity is significantly greater in spontaneously arising PyMT breast cancers, which develop within ductal tissue, than in orthotopic PyMT tumors, which develop as a ball of tumor cells outside the normal ductal architecture (34). This is an important consideration as the former reflects the natural history of human breast cancer and because ductal macrophages are believed to be the TRM population that contribute to breast cancer development (33, 34). Secondly, concomitant expression of M1 and M2 markers in various TAM subtypes across several studies indicates that the concept of anti-tumoral M1-like and pro-tumoral M2 TAMs-like is well and truly outdated (80, 81).

### 3.3 Therapeutic targeting of TAMs

The broad range and prolonged effects of the tumor promoting activities of TAMs have made them obvious therapeutic targets. In addition, TAMs interfere with patient responses to chemotherapy and radiotherapy as cytotoxic therapies result in increased macrophage infiltration or altered TAM behavior (112–115). One particular mechanism of therapeutic interference was revealed when paclitaxel treatment of PyMT mice was shown to increase tumor cell secretion of CSF-1 and IL-34 and addition of a CSF-1R inhibitor to the treatment regime reduced tumor growth and rates of pulmonary metastasis (116). TAMs can also interfere with treatment response by directly secreting growth and angiogenic factors to maintain tumor cell survival and, through their promotion of immune evasion, they are potent inhibitors of responses to immunotherapies (117, 118).

Because macrophages depend on CSF-1 for survival, TAM drug development was initially focused on depleting them through inhibition of the CSF-1R, either by small molecule inhibitors or antibodies targeting the CSF-1/CSF-1R axis. CSF-1R inhibition reduces tumor growth in several mouse models of cancer, including PyMT-driven mammary cancer, cervical cancer, glioblastoma and melanoma (119−121). Surprisingly, CSF-1R inhibition in the glioblastoma model does not reduce TAM numbers but alters their phenotype from pro-tumoral to anti-tumoral in response to tumor secretion of granulocyte macrophage (GM)-CSF (120). However, while a CSF-1R blocking antibody is clinically useful in tenosynovial giant cell tumors driven by constitutive synovial CSF-1 production, clinical trials of CSF-1R inhibitors as single agents have proven disappointing (118, 122). Moreover, long term administration of these agents also depletes TRMs with adverse consequences and there is evidence that TAMs can also promote cytotoxic T cells responses such that wholesale TAM ablation can reduce anti-tumoral immunity (118, 123).

Hence, rather than eliminating TAMs altogether, attention turned towards blocking monocyte recruitment to tumors or reprogramming TAM behavior within tumors (111, 118). Monocyte recruitment can be inhibited by targeting either CCL2 or its receptor, CCR2. However, a CCL2 neutralizing antibody did not show any clinical benefit in a trial of advanced solid cancers and more recent CCL2 inhibitor trials have also been disappointing, perhaps because monocytes continue to be recruited by alternative chemokines (118, 124). Concerningly, interruption of CCL2 inhibition in several metastatic mouse models of breast cancer models appears to accelerate bone marrow monocyte release and increase the rates of metastasis and death, suggesting that caution should be exercised with CCL2/CCR2 inhibitor development (125).

Thus, a number of TAM drug development programs have been directed towards targeting specific pro-tumoral behaviors of TAMs. For example, tumor cells express a ‘don’t eat me signal’ CD47, which interacts with TAM-expressed signal regulatory protein (SIRP)α to stop tumor cells from being phagocytosed (126). Antibodies targeting CD47 enhance tumor cell phagocytosis to reduce tumor growth in xenograft models and a number of clinical trials of anti-CD47 antibodies and small molecule inhibitors have produced good results in lymphomas (127). However, their therapeutic use has been limited by adverse hematological effects (127). Although a range of other TAM-reprogramming therapeutics are currently under development, they are not reviewed here (118, 128). Considering the lethal contribution of TAMs to tumor invasion and metastasis, it is worth examining whether TAM migration could be targeted to inhibit tumor invasion. As outlined earlier, TAMs depend on the CSF-1/CSF-1R axis not only to stimulate their migration via PI3K p110δ/AKT and HCK signaling but also to acquire the molecular machinery supporting macrophage interstitial migration. There are several clues that macrophage motility plays an important role in tumor growth and invasion and that targeting macrophage motility might be a useful therapeutic approach. Firstly, selective inhibition of either PI3K p110δ or HCK with idelalisib or RK20449 respectively shut down both motility and matrix degradation in macrophages *in vitro* (65, 70, 72). These findings can be extended in a complex *in vitro* co-invasion assay using mammary tumor spheroids pre-infiltrated with macrophages, where tumor cell invasion is completely blocked by inhibition of macrophage motility signaling (36, 72). Finally, both RK20449 or acalisib, another PI3K p110δ inhibitor, produce striking reductions in orthotopic PyMT mammary tumor growth *in vivo* (Murrey et al., manuscript submitted) (129). Inhibition of macrophage motility is, therefore, an alternative and potentially powerful approach to therapeutically target the invasion and metastasis-promoting behavior of TAMs.

## 4 Concluding comments

We now understand that macrophages have critical immune and non-immune functions in the body, beginning in embryogenesis and lasting throughout life. Embryonic macrophages infiltrate into every tissue and organ system where they rapidly differentiate into highly specific TRMs that contribute to normal development and then actively monitor the local environment for signs of perturbation of homeostasis. In order to undertake regular and comprehensive tissue surveillance as well as activate repair mechanisms, TRMs either move or extend dynamic dendritic branches to explore their regions of responsibility. CSF-1 is the most important cytokine regulating TRM survival, differentiation and migration. Tumors, which are aberrant organs, secrete CSF-1 to recruit monocyte-derived macrophages as well as subvert the normal housekeeping activities of TRMs to promote tumor invasion and metastasis, angiogenesis, immunosuppression and resistance to cytotoxic therapies. Therefore, it is not surprising that TAMs form a compelling target for drug development in the treatment of cancer, especially in combination with other therapies. While blockade of the CSF-1R itself has not proven helpful in the clinic except for tenosynovial giant cell tumors, the many deleterious behaviors of TAMs can be specifically targeted, including the particularly dangerous effect they have on tumor invasion and metastasis.
